# The extracellular matrix glycoprotein tenascin‐X regulates peripheral sensory and motor neurones

**DOI:** 10.1113/JP276300

**Published:** 2018-07-18

**Authors:** Rubina Aktar, Madusha Peiris, Asma Fikree, Vincent Cibert‐Goton, Maxim Walmsley, Iain R. Tough, Paulo Watanabe, Eduardo J. A. Araujo, Sahar D. Mohammed, Jean‐Marie Delalande, David C. Bulmer, S. Mark Scott, Helen M. Cox, Nicol C. Voermans, Qasim Aziz, L. Ashley Blackshaw

**Affiliations:** ^1^ Blizard Institute Queen Mary University of London London UK; ^2^ Institute of Psychiatry, Psychology and Neuroscience Kings College London London UK; ^3^ Department of Histology, Centre for Biological Sciences State University of Londrina Brazil; ^4^ Department of Neurology Radboud University Medical Centre Nijmegen Netherlands

**Keywords:** Colonic motility, Colonic hypersensitivity, Enteric neurones

## Abstract

**Key points:**

- Tenascin‐X (TNX) is an extracellular matrix glycoprotein with anti‐adhesive properties in skin and joints. Here we report the novel finding that TNX is expressed in human and mouse gut tissue where it is exclusive to specific subpopulations of neurones.
- Our studies with TNX‐deficient mice show impaired defecation and neural control of distal colonic motility that can be rescued with a 5‐HT_4_ receptor agonist. However, colonic secretion is unchanged. They are also susceptible to internal rectal intussusception. Colonic afferent sensitivity is increased in TNX‐deficient mice. Correspondingly, there is increased density of and sensitivity of putative nociceptive fibres in TNX‐deficient mucosa.
- A group of TNX‐deficient patients report symptoms highly consistent with those in the mouse model.
- These findings suggest TNX plays entirely different roles in gut to non‐visceral tissues – firstly a role in enteric motor neurones and secondly a role influencing nociceptive sensory neurones
- Studying further the mechanisms by which TNX influences neuronal function will lead to new targets for future treatment.

**Abstract:**

The extracellular matrix (ECM) is not only an integral structural molecule, but is also critical for a wide range of cellular functions. The glycoprotein tenascin‐X (TNX) predominates in the ECM of tissues like skin and regulates tissue structure through anti‐adhesive interactions with collagen. Monogenic TNX deficiency causes painful joint hypermobility and skin hyperelasticity, symptoms characteristic of hypermobility Ehlers Danlos syndrome (hEDS). hEDS patients also report consistently increased visceral pain and gastrointestinal (GI) dysfunction. We investigated whether there is a direct link between TNX deficiency and GI pain or motor dysfunction. We set out first to learn where TNX is expressed in human and mouse, then determine how GI function, specifically in the colon, is disordered in TNX‐deficient mice and humans of either sex. In human and mouse tissue, TNX was predominantly associated with cholinergic colonic enteric neurones, which are involved in motor control. TNX was absent from extrinsic nociceptive peptidergic neurones. TNX‐deficient mice had internal rectal prolapse and a loss of distal colonic contractility which could be rescued by prokinetic drug treatment. TNX‐deficient patients reported increased sensory and motor GI symptoms including abdominal pain and constipation compared to controls. Despite absence of TNX from nociceptive colonic neurones, neuronal sprouting and hyper‐responsiveness to colonic distension was observed in the TNX‐deficient mice. We conclude that ECM molecules are not merely support structures but an integral part of the microenvironment particularly for specific populations of colonic motor neurones where TNX exerts functional influences.

## Key points

- Tenascin‐X (TNX) is an extracellular matrix glycoprotein with anti‐adhesive properties in skin and joints. Here we report the novel finding that TNX is expressed in human and mouse gut tissue where it is exclusive to specific subpopulations of neurones.
- Our studies with TNX‐deficient mice show impaired defecation and neural control of distal colonic motility that can be rescued with a 5‐HT_4_ receptor agonist. However, colonic secretion is unchanged. They are also susceptible to internal rectal intussusception. Colonic afferent sensitivity is increased in TNX‐deficient mice. Correspondingly, there is increased density of and sensitivity of putative nociceptive fibres in TNX‐deficient mucosa.
- A group of TNX‐deficient patients report symptoms highly consistent with those in the mouse model.
- These findings suggest TNX plays entirely different roles in gut to non‐visceral tissues – firstly a role in enteric motor neurones and secondly a role influencing nociceptive sensory neurones
- Studying further the mechanisms by which TNX influences neuronal function will lead to new targets for future treatment.

## Introduction

The extracellular matrix (ECM) provides a basic intercellular scaffold critical in tissue development, differentiation, signalling and homeostasis. The ECM comprises macromolecules including the glycoprotein tenascin‐X (TNX) (Zweers *et al*. 2003). TNX contributes to ECM around connective tissues within skin/joints through anti‐adhesive interactions with collagen (Valcourt *et al*. 2015). Monogenic TNX deficiency causes hypermobility of skin including tissue fragility and musculoskeletal pain, a phenotype similar to hypermobile Ehlers‐Danlos syndrome (hEDS) (Burch *et al*. 1997; Hakim & Grahame, 2003). Interestingly there is a high (39%) representation of hEDS amongst patients with functional gastrointestinal (GI) disorders (FGIDs) (Fikree *et al*. 2015). A recent study in classical, vascular and hypermobile EDS showed 56% of patients had GI manifestations (Nelson *et al*. 2015). Common symptoms were abdominal pain (56.1%), nausea (44.3%), constipation (42.4%) and FGID symptoms (39%) with symptom severity correlating with more severe hEDS phenotype (Fikree *et al*. 2014). Physiological studies demonstrate hEDS patients have colonic dysmotility, with 28.3% of hEDS patients exhibiting delayed colonic transit (Nelson *et al*. 2015). However, the mechanisms underlying changes to colonic transit in hEDS are unknown.

Recently, TNX deficiency has been classified as a distinct subgroup (classical‐like EDS) within the EDS spectrum disorders (Malfait *et al*. 2017) and GI manifestations are particularly common in these patients. For instance, case studies in TNX‐deficient patients report constipation, rectal prolapse, abdominal pain, diverticulosis and hiatal hernia (Schalkwijk *et al*. 2001; Lindor & Bristow, 2005). However, the cellular mechanisms mediating GI symptoms are yet to be described. TNX‐deficient mice show musculoskeletal and cutaneous features of hEDS (Mao *et al*. 2002) while other aspects of physiology remain uninvestigated.

Given that hindgut symptoms are common in hEDS and TNX‐deficient patients, we hypothesized that TNX is needed for sensorimotor function of the colon. Our search for a mechanistic link required an understanding of both anatomical expression and the functional role of TNX in the colon. We characterized colocalization of TNX in mouse and human gut with neuronal markers. To understand the functional role of TNX, it was important to perform detailed assessment of colon function. Colonic function requires the integration of multiple complex pathways including coordinated action of enteric neurons and smooth muscle cells, and input from external sources (Sarna, 1993). These influence myogenic and enteric neuronal regulation to modify gut contractions in response to the environment (Sarna, 2010). To assess the overall role of TNX in the colon we studied: gross anatomy and defecatory function (Page *et al*. 2004); colonic motor coordination *in vitro* (Fraser *et al*. 1997); activity of colonic splanchnic nerves using *in vitro* afferent electrophysiology (Hockley *et al*. 2017); and the ability of the colonic epithelium to secrete ions *in vitro* (Cox *et al*. 2001). Finally, we assessed GI symptoms in patients with TNX deficiency.

We show in both mouse and human gut that TNX expression is associated not with connective tissue, but with enteric neurones. In the genetically confirmed TNX‐deficient patients, we assessed if GI symptoms are similar to those reported in case studies, and in the hEDS subgroup (Demirdas *et al*. 2016). To understand the mechanistic role of TNX, we used a knockout mouse (Mao *et al*. 2002). Protein expression and the functional role of TNX in the GI tract have not been investigated previously, except one study showing mRNA expression in porcine stomach and colon (Geffrotin *et al*. 1995).

We provide evidence for the first time that an ECM molecule – TNX – has a role in colonic function. Gut function is disrupted by the increased incidence of prolapse in TNX‐deficient mice and humans. Colonic motility is attenuated, particularly in the distal segment, which is reversible pharmacologically. Colonic nociceptive afferents are hypersensitive in the absence of TNX, and there is increased sensory neuronal sprouting. The expression and functional outcomes in the TNX‐deficient mouse match closely the reported symptoms in TNX‐deficient patients. Based on the expression, functional and patient symptom data we provide a role for TNX in maintaining normal colonic function. Overall, our studies have important implications for understanding the role and exact mechanism of how ECM components influence gut function in health and disease.

## Methods

### Ethical approval

Full‐thickness non‐pathological colonic tissue was obtained from cancer patients (>10 cm away from tumours) undergoing colonic resections using approved Human Research Ethics from Bart's and London NHS Trust with informed consent (NREC 09/H0704/2). TNX‐deficient patients gave written informed consent prior to completing the questionnaires.

All studies were completed according to the animal ethics policy and the study is in compliance with guidelines set out by Grundy (2015). All mice used in these studies were killed by asphyxiation using carbon dioxide in accordance with the UK Home Office (Schedule 1, Animals Act 1986) for all experimental procedures. All human questionnaire studies were completed in accordance with the *Declaration of Helsinki*.

### Mouse tissue

All mice used in this study originated from parent mice with a C57BL/6N background and were donated by Professor Manuel Koch (University of Cologne). From this group, TNX‐KO was created by targeting the 5′ end of *TNXB* gene, replacing the first five coding exons with lacZ and a neomycin resistance cassette (Mao *et al*. 2002). Knockout (KO) and wild‐type mice showed similar growth and were of similar weight at the time of the study (WT: 19.9 ± 0.8 g and KO: 20.8 ± 1.1 g). All mice were reared and transported under conditions specified in the UK's Animal Welfare Act 2006. Mice aged between 10 and 12 weeks were used and were killed by a rising concentration of CO_2_ asphyxiation (Schedule 1, Animals Act 1986, UK Home Office).

### Immunohistochemistry

Immunolabelling with rabbit polyclonal TNX (1:200, Santa Cruz, sc‐25717, Santa Cruz, CA, USA), calretinin (1:500, Swant, CG1 and 6B3, Marly, Switzerland), calcitonin gene‐related peptide (CGRP) (1:400, Abcam, ab36001, Cambridge, MA, USA; and Thermo Fisher, ABS026‐05‐02, Waltham, MA, USA), choline acetyltransferase (ChAT) (1:400, Millipore, AB144P, , Billerica, MA, USA), neuronal nitric oxide synthase (NOS) (1:400, Abcam, ab1376), protein gene product 9.5 (PGP9.5) (1:500, Agilent/DAKO‐Z5116, , Santa Clara, CA, USA) was assessed in human‐ and mouse‐specific GI regions.

#### Immunohistochemistry process

Tissue was fixed in 4% paraformaldehyde, 10 μm sections and whole mounts were prepared, blocked in universal blocking serum (Dako, Carpinteria, CA, USA) and incubated overnight at 4°C with primary antibodies. Slides/whole mount tissue was then incubated for 1 h at room temperature with AlexaFluor conjugated secondary antibody as appropriate. Slides/whole mounts were then mounted using a coverslip and Vectashield Hardset Mountant conjugated with DAPI (H‐1500) and left to dry before viewing under the microscope at 40×.

### Motility studies

Colonic motility in mice was determined using a multilumen perfusion manometry apparatus (Fraser *et al*. 1997). Spontaneous pressure waves were recorded, and nitric oxide synthase inhibitor [*N*‐ω‐nitro‐l‐arginine (NOLA), 100 μm) and bile acid (deoxycholic acid, 100 μm) induced effects on motility were assessed. Separate experiments with prucalopride (3 μm) addition were performed in each mouse group, and similarly various motility parameters were assessed. Recordings were obtained and analysed manually using medical measurement system (MMS) software.

### Pellet output

Mice were housed overnight in individual cages with a wire bottom to collect faecal pellets over 6 days. The total number of pellets was counted and weighed daily.

### Ussing chamber studies

Submucosal–mucosal sheets were isolated and mounted in Ussing chambers (exposed area of 0.14 cm^2^) continuously bathed in Krebs‐Henseleit buffer as described previously (Hyland *et al*. 2003). The mucosae were voltage clamped at 0 mV (DVC 1000, World Precision Instruments, Stevenage, UK) and short‐circuit current (*I*_Sc_) was recorded continuously. Veratridine (which depolarizes the intact submucosal innervation of the mucosa and causes epithelial anion secretion) was added at 30 μm to each serosal hemi‐chamber and changes in ion transport were measured. Data were acquired using a 1401 and Spike2 software (Cambridge Electronic Design, Cambridge, UK).

### Colonic afferent recording studies

The distal colons from WT and TNX‐KO mice were removed with associated lumbar splanchnic nerves and activity was recorded using previously described methods (Hockley *et al*. 2017). Once a stable recording was achieved (approx. 20 min), a ramp distension was performed by blocking luminal perfusion out‐flow of the cannulated colon producing noxious pressures known to both evoke pain behaviours *in vivo* in mice and robustly activate all known afferent mechanoreceptors (0–80 mmHg). After 10 min, four successive rapid phasic ramp distensions (0–80 mmHg, 60 s at 9 min intervals) were performed by switching luminal perfusion outflow of the cannulated colon to an 80 mmHg end pressure. The tissue was superfused with carbogenated (95% O_2_, 5% CO_2_) Krebs buffer (6 ml/min; 32–34°C; pH 7.4; 124 mm NaCl, 4.8 mm KCl, 1.3 mm NaH_2_PO_4_, 1.2 mm MgSO_4_.7H_2_O, 2.5 mm CaCl_2_, 11.1 mm glucose, 25.0 mm NaHCO_3_). Compliance was measured which is the total time it takes to reach 80 mmHg intraluminal pressure from the moment the outflow is closed, multiplied by the rate of intraluminal perfusion (0.1 ml/min). Data were analysed using Spike 2 software.

### Patient genotyping

Eleven patients (three males and eight females) identified from Radboud University Medical Centre took part and were genotyped for *TNXB* (Schalkwijk *et al*. 2001). Mutation analysis was performed using next‐generation sequencing (NGS) testing for the *TNXB* DNA mutation, as previously described (Demirdas *et al*. 2016). Additionally, serum samples were analysed for TNX glycoprotein using ELISA with rabbit anti‐TNX.

### GI symptom questionnaire

Patients with TNX deficiency completed a Flemish version of the validated Gastrointestinal Symptom Rating Scale (GSRS) (Svedlund *et al*. 1988). Questionnaire responses were translated and scored for five different domains – reflux, abdominal pain, constipation, indigestion and diarrhoea. Scores ranged from 1 (no symptoms) to 7 (unbearable symptoms). GSRS scores for abdominal pain and lower GI symptoms for constipation and diarrhoea were analysed in TNX‐deficient patients compared to a reference Swedish population of similar age and sex (Dimenas *et al*. 1996).

### Experimental design and statistical analysis

#### Immunohistochemistry

Quantitative assessment of immunohistochemistry images were taken from *n* = 9 WT *vs. n*= 9 KO (five females and four males in both groups) of which five fields of view were captured from each mouse. Therefore 45 images per neuronal marker with TNX were then analysed. To determine whether a neuron is positive we used an approximate threshold of 20% above background. The background was defined as 0% which was black, while the brightest area in the image was 100%. Additionally, two blinded researchers completed the neuronal counts according to the criteria mentioned. Controls with no primary antibody were performed with each run of immunohistochemistry to confirm the specificity of the primary antibody, which resulted in no fluorescence above background. Western blot with this antibody in humans revealed a band at the predicted weight of 268 kDa. Moreover, TNX was absent from all TNX‐KO gut tissue. Images were obtained using Metamorph software on an Olympus MM Leica (sections) or Zen software on a Zeiss LSM 710 or Zeiss LSM 880 (confocal imaging whole‐mounts) at 40× magnification. Statistical analysis was performed using an unpaired *t*‐test for each marker and a *P*‐value of < 0.05 was deemed significant.

#### Colonic motility

A total of 11 WT and 11 KO mice (six females and five males in both groups) were used to measure spontaneous contractile activity of the colon. Experiments using NOLA and bile acid–deoxycholic acid were completed separately; *n* = 8 WT *vs. n* = 9 KO mice (four females and four males in WT and five females and four males in KO group) for NOLA experiments and *n* = 5 WT *vs. n* = 5 KO mice (three females and two males in both groups) for bile acid experiments. Pellet output studies used *n* = 9 WT *vs. n* = 9 KO mice (five females and four males in both groups) over 6 days and pellets were counted daily. Separate prucalopride experiments in *n* = 6 WT *vs. n* = 6 KO mice (four females and two males in both groups) were also performed. Data from all motility studies were statistically analysed with an unpaired *t‐*test to compare each colonic region. For pellet output studies significance was calculated using an unpaired *t‐*test. Individual data were plotted for all data sets and a *P*‐value of < 0.05 was deemed significant.

#### Colonic secretion

A total of *n* = 11 WT *vs. n* = 9 KO mice (six females and five males in WT and five females and four males in KO) were used. Statistical analysis was performed with an unpaired *t‐*test to compare each colonic region in both groups. All individual data were plotted and a *P*‐value of < 0.05 was deemed significant.

#### Colonic afferent recording

A total of *n* = 3 WT *vs. n* = 3 KO mice (three females in both groups) were used. Statistical analysis was performed using an unpaired *t‐*test. Individual data were plotted and a *P*‐value of < 0.05 was deemed significant.

#### Patient questionnaires

Eleven patients (three males and eight females) with TNX deficiency were used and compared to a reference Swedish population of *n* = 2162. The reason for the small sample size in the TNX‐deficient group was due to a small number of patients known to have TNX deficiency since it is uncommonly measured. Questionnaire data are shown as mean values with standard deviation. Statistical analysis was performed using an unpaired Student's *t‐*test for each symptom (*P *< 0.05).

All data are expressed as individual data points and variability within the data was represented using 95% confidence intervals of the mean. Statistical analysis was performed using GraphPad Prism (v.7.02, GraphPad Software, Inc., La Jolla, CA, USA).

## Results

### TNX localization in mouse and human GI tract

**Figure 1:**
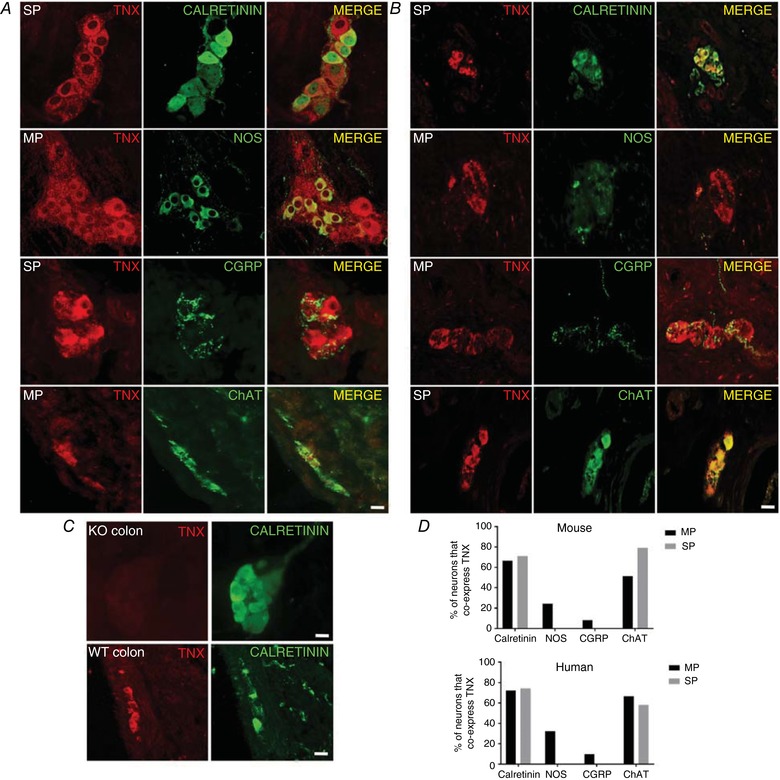
TNX expression in mouse and human neural structures Representative immunohistochemical images taken from whole mounts and sections of WT mouse (*A*) and human distal colon (*B*). TNX‐IR was commonly expressed in calretinin‐positive neurones in the mouse myenteric plexus (MP) (*A*‐merge) and human submucous plexus (SP) (*B*‐Merge), while TNX‐ and NOS‐positive cells in the MP showed few co‐labelled cell bodies in both mouse and human colon (*A* and *B*‐Merge). TNX‐IR and CGRP expression is mutually exclusive but in close apposition whereby CGRP‐positive fibres surround TNX‐positive myenteric neurones in mouse and human colon (*A* and *B*‐Merge). TNX specificity is confirmed by the absence of TNX in TNX‐KO mouse colon while calretinin‐positive neurones are present in the submucous plexus (*C*). TNX was not found in the mucosa while calretinin fibres are present (*C*). *D*, average number of neuronal cell bodies per field of view (fov) that co‐labelled TNX plus calretinin/CGRP/NOS and ChAT in SP and MP in mouse and human colon respectively. TNX co‐expressed significantly more with calretinin (55.5%) and ChAT (51.6%) than NOS in mouse MP neurones, with none in SP and only one neuron in MP. In human colon TNX‐IR co‐expressed significantly more with ChAT in the MP (100%) and SP (52.58) than NOS (*D*). CGRP did not co‐label with any TNX‐IR cell bodies in the SP and only one in MP in mouse and human (*D*). Images on the first three panels (*A*) were obtained on a confocal microscope and were taken from a compressed optical slice in a *z* stack. All other images were obtained from sections. Data shown as percentages of total neurons positive for TNX and respective markers. Statistical analysis was performed using an unpaired Student's *t‐*test for each region, *P* < 0.05. Scale bar in all panels = 50 μm.

TNX‐immunoreactivity (IR) in mouse distal (Fig. 1 *A*) and human descending colon (Fig. 1 *B*) was found specifically within myenteric and submucous plexus neurones and not within connective tissue. Neuronal counts within the mouse submucous and myenteric plexuses revealed that TNX‐IR extensively co‐localized with calretinin‐IR, and ChAT‐IR (markers for excitatory enteric motorneurones) (Mazzuoli & Schemann, 2012), but only in a few NOS‐IR (inhibitory) enteric neurones (Fig. 1 *E*) in both mouse and human colon. The nociceptive afferent marker CGRP‐ (Sharrad *et al*. 2015) and TNX‐IR were in close apposition in mouse (Fig. 1 *A*) and human colon (Fig. 1 *B*), but not directly co‐localized. CGRP‐positive nerve fibres surrounded myenteric ganglia but did not project within ganglia containing TNX‐IR neuronal cell bodies (Fig. 1 *A* and *B*). CGRP‐IR was lowest in neuronal counts of cell bodies co‐labelling with TNX (Fig. 1 *C* and *E*). Quantitative polymerase chain reaction (qPCR) analysis showed TNX was present at the RNA level in mouse colon (data not shown).

The location of TNX, revealed by confocal optical sections, suggested that it was intracellular within neuronal cell bodies (Fig. 1). TNX expression was found to partially overlap with calretinin, an intracellular marker (Fig. 1 *A*). TNX‐IR was absent from the mucosa of all tissue in mouse (Fig. 1 *C*) and human gut (data not shown). Importantly, in TNX‐KO distal colon, calretinin‐IR myenteric neurones were still observed, but with no TNX staining as expected, confirming the specificity of the antibody (Fig. 1 *D*). In addition, unlike TNX, tenascin C (TNC) was not found in enteric neurones and was instead found in muscle layers and connective tissue within the colon (data not shown).

### Spontaneous colonic migrating motility in mice

**Figure 2:**
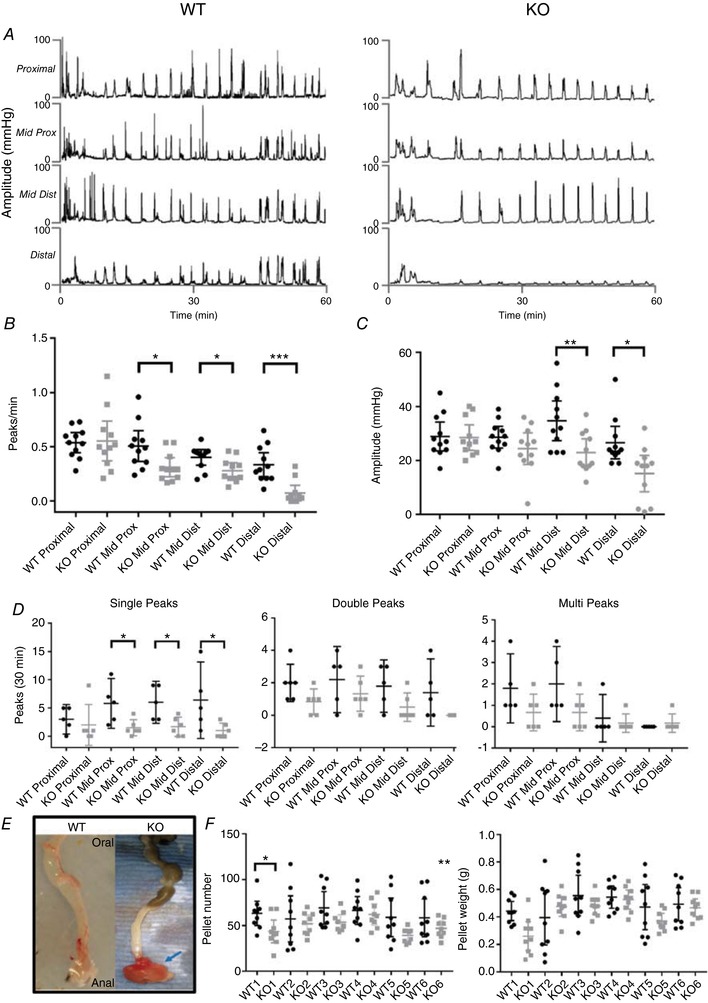
Colonic motility and pellet output in WT *vs*. TNX‐KO mice Manometry studies showed a reduction in the number and amplitude of spontaneous pressure waves in TNX‐KO mice (*A*) (*n* = 11 WT *vs. n* = 11 KO). The number of peaks/min was significantly reduced in the mid‐proximal (*P = *0.0475), mid‐distal (*P = *0.0482) and distal colon (*P = *0.0031) (*B*) and amplitude of pressure waves was significantly reduced in the mid‐distal (*P = *0.0069) and distal colon only (*P = *0.021) in TNX‐KO mice (*C*). The total number of spontaneous single peaks over 30 min showed a significant reduction in all colonic regions of TNX‐KO mice except for the proximal colon (*D*). There was significant change in the number of double and multi‐peaks in all colonic regions in WT *vs*. KO mice (*D*). WT colon and rectum were normal whereas in the TNX‐KO the rectum shows intussusception, as indicated by arrow (*E*). This was observed in 12% of 38 TNX‐KO mice. The number of faecal pellets per day showed a significant decrease in TNX‐KO mice (*P = *0.0002) although there was no significant change in pellet weight (*F*) (*n* = 9 WT *vs. n* = 9 KO). Data are shown as individual values with standard deviation. Statistical analysis was performed using an unpaired Student's *t* test for each region, *P* < 0.05.

**Figure 3:**
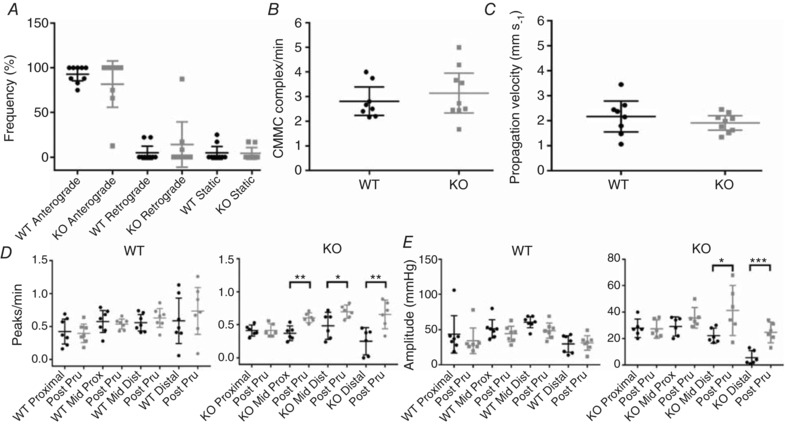
CMMCs and the effect of prucalopride Detailed analysis on the finer control of the colon, for example in colonic migrating motor complexes (CMMCs), showing that CMMC direction (*A*), frequency (*B*) and propagation velocity (*C*) did not change in either group. Addition of 3 μm prucalopride (Pru) significantly increased the number of peaks in TNX‐KO mid‐proximal, mid‐distal and distal colon (*P = *0.0031, *P* < 0.0001 and *P < 0*.0001, respectively) (*D*) (*n* = 6 WT *vs*. *n* = 6 KO). The amplitude of contractions was also restored by the addition of prucalopride in the mid‐distal (*P = *0.0262) and distal colon (*P = *0.0007) (*E*). Data are shown as individual values with standard deviation. Statistical analysis was performed using an unpaired Student's *t* test for each region (*P* < 0.05).

The localization of TNX in enteric plexuses strongly suggested a role in neural control of motility and secretion. We investigated this by determining the disruption in bowel function of TNX‐deficient mice, in which we studied complex neural control of colonic motor function *in vitro*. The rate and amplitude of spontaneous pressure waves were significantly reduced in TNX‐KO mice compared to WT, particularly in the distal colonic segments (Fig. 2 *A*), and therefore the strength of contractions was weakest in these regions (Fig. 2 *C*). Peak type differentiation analysis showed a reduction in single peaks but not double or multiple peaks (Fig. 2 *D*). The addition of the neuronal prokinetic 5‐HT_4_ receptor ligand prucalopride (3 μm) rescued the impaired motility by increasing the number of contractions in the mid‐proximal, mid‐distal and distal colon of TNX‐KO mice but showed no changes in WT mice (Fig. 3 *D* and *E*). Analysis of colonic migrating motor complexes (CMMCs) showed no difference in CMMC direction (Fig. 3 *A*), frequency (Fig. 3 *B*) or propagation velocity (Fig. 3 *C*) between WT and TNX‐KO. The effect of NOS inhibition with NOLA (Brierley *et al*. 2001) and activation of neural inhibition via TGR5 using deoxycholic acid (Bunnett, 2014) was similar in both genotypes (data not shown). NOS inhibitors and deoxycholic acid were used to test the role of TNX in inhibitory pathways to smooth muscle because NOS and TGR5 are involved in the NO releasing pathways involved in inhibiting colonic contractility.

### Internal rectal intussusception and faecal output in mice

Based on our observations of disordered motility in TNX‐deficient mice, it would follow that disruption of bowel habit would also occur *in vivo*, so we investigated if the loss of TNX affects defecatory function. In total, 12% of 38 TNX‐KO mice had an internal rectal intussusception (defined as a partial eversion of a region of the bowel), whereas WT had none (Fig. 2 *E*). Overall faecal pellet output decreased significantly in TNX‐KO mice, particularly on day 1 (*P* < 0.001), but there was no change in pellet weight (Fig. 2 *F*).

### Neurally evoked secretory responses in mouse colon

**Figure 4:**
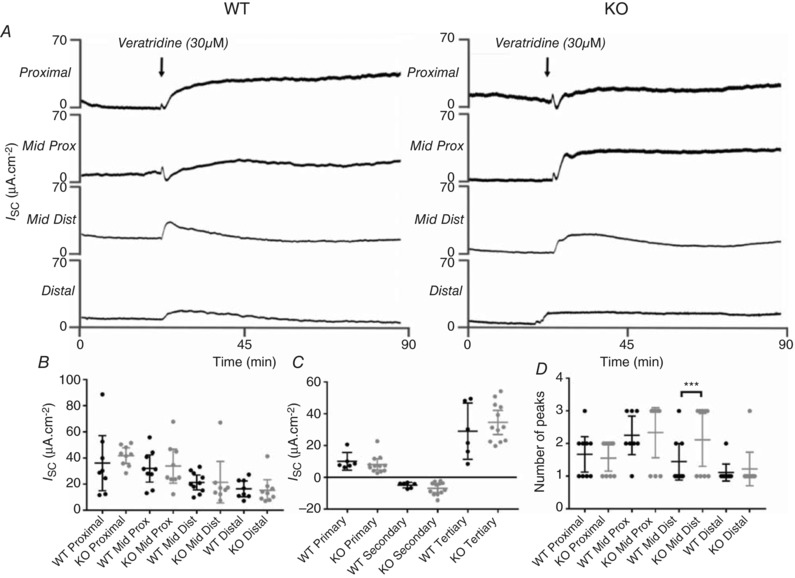
Colonic mucosal ion transport responses in WT and TNX‐KO mice Representative traces showing responses to veratridine in designated colonic tissues from WT *vs*. TNX‐KO mice (*A*). At 20 min veratridine was added and changes in *I*_Sc_ were recorded for 90 min. No difference in veratridine‐induced responses was seen in the colonic regions of WT and TNX‐KO mice (*B*). Overall colonic response to veratridine also showed no change at initial peak (over 1 min, i.e. primary), second dip (within 2 min, i.e. secondary) or tertiary peak (10 min) (*C*). The number of mice with triphasic (i.e. an initial peak, followed by a drop below baseline followed by an overall increase in secretion) responses to veratridine was significantly increased in KO colon compared to WT in the mid‐distal colon (*P = *0.0194) (*D*) (*n* = 11 WT *vs. n* = 9 KO). Data are shown as individual values with standard deviation. Statistical analysis was performed using an unpaired Student's *t test* for each region (*P* < 0.05).

As described earlier, TNX localization in submucous neurones suggested a role in neural control of mucosal ion transport. This was measured as changes in *I*_Sc_ after addition of the neuronal depolarizing agent veratridine, which induces rapid, sustained increases in *I*_Sc_ by activating neuronal sodium channels (Hyland & Cox, 2005). There were no significant differences in complex time‐dependent changes in *I*_Sc_ between WT and KO in any colonic region following veratridine (Fig. 4 *A* and *B*). The secondary analysis involved measuring initial peak *I*_Sc_ within the first minute, a secondary *I*_Sc_ decrease after initial peak within 2 min and a tertiary peak *I*_Sc_ within the first 10 min of exposure to veratridine, which showed no significant differences between WT and TNX‐KO (Fig. 4 *C*).

Finally, the number of monophasic (increase above baseline), biphasic (decrease below baseline followed by an increase above baseline) and triphasic *I*_Sc_ responses (initial increase above the baseline, followed by a decrease below baseline, finishing with the overall maximum peak) were analysed following veratridine. The overall number of veratridine response phases in TNX‐KO increased significantly (*P* < 0.0001) in particular, mid‐distal colon (*P = *0.0005), indicating subtle differences in neural influences on mucosal ion transport in this region (Fig. 4 *D*).

### Sensory fibre density in TNX‐KO colonic mucosa

**Figure 5:**
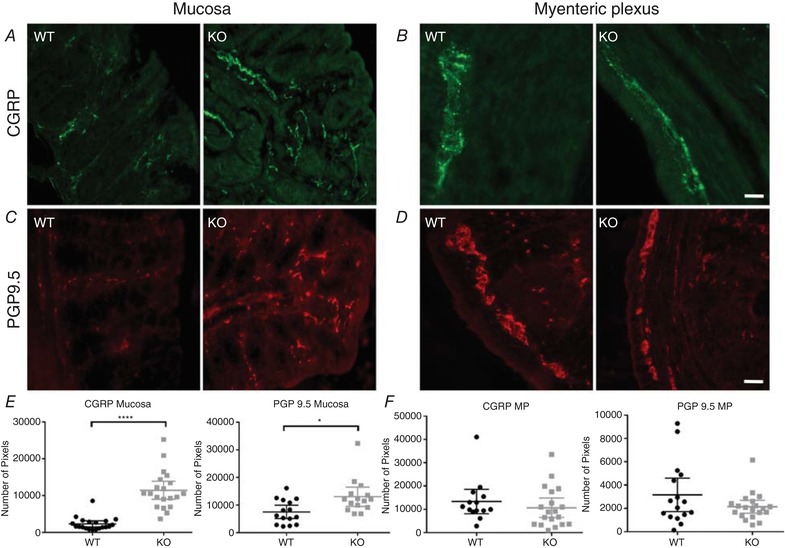
Neuronal sprouting in TNX‐KO mucosa Representative immunohistochemical images taken from sections of WT mouse colonic mucosa (*A*, *C*) and myenteric plexus (*B*, *D*). CGRP‐IR is shown in green (*A*) where there was a threefold proliferation of CGRP‐IR nerve endings in the colonic mucosa of TNX‐KO mice (*P* < 0.0001) (*E*). This was also observed with PGP9.5 in red (*C*), although this increase was smaller for PGP‐IR endings (*P* = 0.0224) (*E*). No changes were seen in either marker in the myenteric plexus (*P = *0.3950, *P* = 0.1368) (*F*). Data are shown as individual values with standard deviation. Statistical analysis was performed using an unpaired Student's *t* test (*P* < 0.05). Scale bar in all panels = 20 μm.

There was no co‐expression of the nociceptor marker CGRP with TNX, indicating no direct involvement in peripheral nociception. However, we noticed upon anatomical examination of these fibres that there was a threefold increase in CGRP‐IR nerve endings in the colonic mucosa of TNX‐KO mice (Fig. 5 *A* and *E*) and a smaller increase in PGP‐IR endings (Fig. 5 *C* and *E*). No changes were observed for either marker in endings surrounding the myenteric plexus (Fig. 5 *B*, *D* and *F*). Therefore, there is a proliferation predominantly of nociceptive nerve fibres in TNX‐KO mucosa.

### Colonic afferent sensitivity in mice

**Figure 6:**
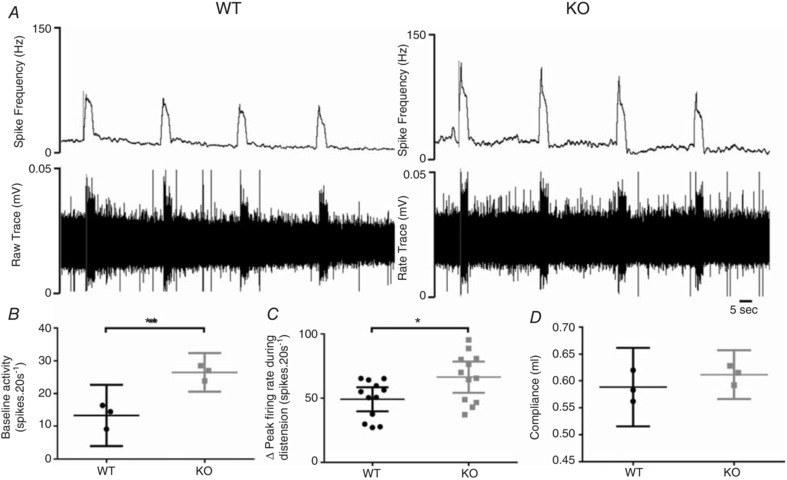
Increased colonic afferent sensitivity in TNX‐KO distal colon Nerve activity from colonic afferent units innervating the distal colon in WT *vs*. TNX‐KO. Nerve activity displayed by spike frequency (Hz) and raw trace (mV) in WT *vs*. TNX‐KO (*A*). Baseline splanchnic nerve activity is significantly increased in TNX‐ KO (*B*) (*P = *0.0069) as well as average peak firing rate during distension (*C*) (*P = *0.0384) without changes in colonic compliance (*D*) (*n* = 3 WT *vs. n* = 3 KO). Data are shown as individual values with standard deviation. Statistical analysis was performed using an unpaired Student's *t* test (*P* < 0.05).

The sprouting of CGRP‐positive fibres in TNX‐KO mice suggested a change in afferent function. Therefore, we investigated if there was an alteration in mechanosensory and electrophysiological properties of colonic afferents that lack TNX. Baseline splanchnic afferent nerve activity was significantly increased in TNX‐KO mice (*P* = 0.0069) (Fig. 6 *A*). Phasic responses to distension showed an overall significant increase in TNX‐KO compared to WT (*P = *0.0206). However, no change in colonic compliance was observed (Fig. 6 *D*), suggesting increased afferent sensitivity in TNX‐KO is a property of the endings, not their environment.

### Gastrointestinal symptoms in TNX‐deficient patients

**Figure 7:**
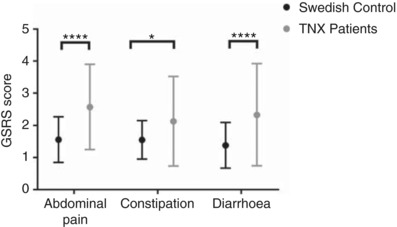
Gastrointestinal symptoms in TNX‐deficient patients and healthy controls The severity of gastrointestinal symptoms assessed as gastrointestinal symptom rating scale (GSRS) scores (means and 95% confidence interval) in patients with TNX deficiency (*n* = 11) and healthy controls (*n* = 2162). Abdominal pain, constipation and diarrhoea were significantly higher compared to Swedish controls (^****^ *P* < 0.0001, ^***^ *P* = 0.0194). Data are shown as mean values with standard deviation. Statistical analysis was performed using an unpaired Student's *t* test for each symptom (*P* < 0.05).

The evidence presented above indicates an important role of TNX in the neural control of the mouse gut, and similar patterns of expression in mouse and human. We were fortunate to be able to access a cohort of genetically confirmed TNX‐deficient patients, in which we investigated its role further. In these patients abdominal pain was increased by 65% compared to controls (*P* < 0.0001). Bowel dysfunctions including constipation (32%, *P* < 0.05) and diarrhoea (64%, *P* < 0.0001) were also increased (Fig. 7). Three of 11 (27%) TNX‐deficient patients assessed in this study reported external rectal prolapse, which is often associated with constipation.

## Discussion

We report TNX is required for neural control of the bowel by a specific subtype of mainly cholinergic enteric neurones, adding to its structural role in somatic connective tissue (Egging *et al*. 2006). TNX also regulates sprouting and sensitivity of nociceptive sensory endings in mouse colon. These findings correlate with symptoms shown by patients and TNX‐deficient mice. Our anatomical and physiological data indicate TNX is important in neural control of colonic sensory and motor function. In mouse and human, we found TNX‐IR exclusively with neuronal structures within myenteric and submucous plexuses. TNX‐KO mice lacked expression of TNX as expected, but had altered sensory innervation, whereby sprouting of CGRP‐IR fibres was observed, indicating an indirect role for TNX in mucosal neural innervation. TNX‐KO mice had disordered colonic motility and this deficit could be restored by increasing cholinergic drive with a 5‐HT_4_ receptor agonist. However, neural control of colonic ion transport was altered only subtly, indicating a specialized role. TNX‐KO mice have increased sensitivity of colonic afferents to noxious distension, probably following from sprouting observed anatomically. In principle, the site of localization corresponds to the site of functional loss that occurs when TNX is deleted.

We expected TNX would be found in association with extracellular collagen fibrils in connective tissue as in mouse skin (Bristow *et al*. 2005). Indeed, TNX was found in dermis (Egging *et al*. 2006). However, in the GI tract, TNX was exclusively expressed in neuronal somata. In mouse and human colon, TNX associated predominantly with ChAT‐ and calretinin‐expressing neurones in submucous and myenteric plexuses, with less labelling in NOS‐positive neurones. This pattern of TNX expression suggests that it has a preferential role in excitatory pathways of smooth muscle and epithelial cells. TNX‐antibody specificity in neurones was confirmed in TNX‐KO mice where labelling was absent. Furthermore TNC was not expressed in neurones but in the connective tissue within colonic smooth muscle. Thus, TNX may play a different role in the gut compared with other tissues – that of a neuronal ECM molecule. Certainly, other tenascins, R and C, are produced by CNS neurones, where they contribute to synaptic plasticity at the synaptic bouton (Kwok *et al*. 2011). In particular, TNR forms a specialized ECM microenvironment called peri‐neuronal nets (PNNs) (Kwok *et al*. 2011). PNNs surround neurones and dendrites in a mesh‐like structure with gaps at synaptic contacts (Celio & Blumcke, 1994) and are involved in synaptic plasticity (Kwok *et al*. 2012). It may be that TNX provides a similar mesh in the enteric nervous system (ENS). However, further expression studies with a range of ECM molecules, such as chondroitin‐sulfate proteoglycans commonly found within PNN structures, are necessary to confirm if an ‘enteric PNN’‐like structure exists.

**Figure 8:**
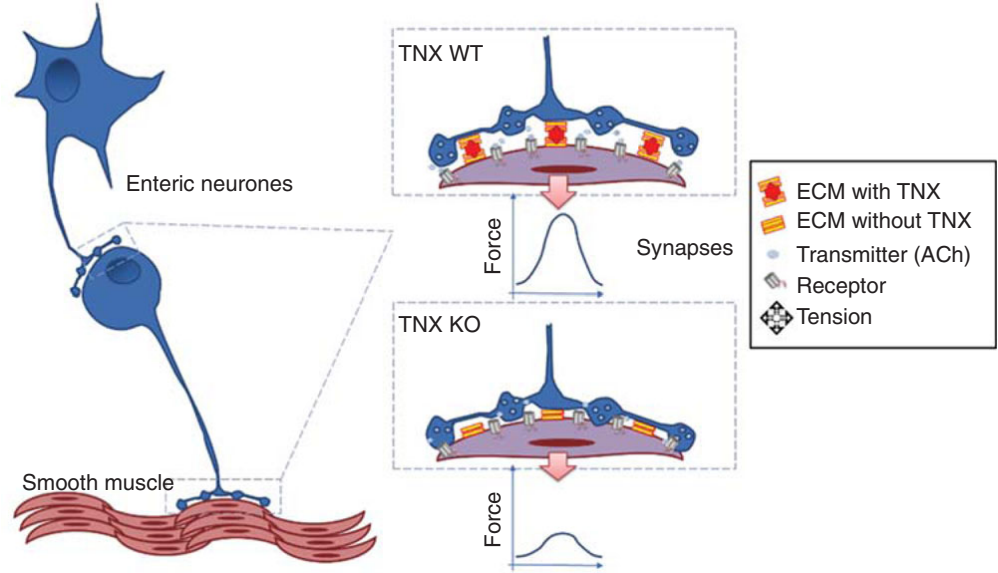
Hypothesized anti‐adhesive mechanisms by which TNX supports the motor function In the colon, TNX acts to maintain correct orientation and spacing of synaptic contacts at neuronal and neuromuscular junctions. Thus, the release of neurotransmitter is able to reach the postsynaptic receptor and engage effectively with it, giving rise to an optimal contractile response to neural activation. Without TNX, these junctions are disorganized, possibly by being too narrow, and fail to allow efficient neurotransmission.

In light of the expression data, we hypothesized that TNX‐KO mice would have a functional deficit within the cholinergic pathway mediating motility. Indeed, TNX deletion caused a significant reduction in the occurrence of spontaneous pressure waves that was most apparent in the distal colon. However, the coordination of contractions showed no overt change, which may signify a role in the final cholinergic motorneurones rather than the integrative interneurones, which are more likely to coordinate the organization/migration of CMMC throughout the colon (Spencer & Bywater, 2002). This concept is shown schematically in Fig. 8, where defective anti‐adhesive properties in TNX deficiency are hypothesized to lead to reduced neurotransmission. NOS inhibition had similar effects in WT and TNX‐KO, as did activation of TGR5 with bile salt indicating TNX has little role in inhibitory pathways to smooth muscle. Cholinergic blockade within the ganglion or at the neuromuscular junction both abolish CMMC (Brierley *et al*. 2001), so it was not possible to test pharmacologically the extent of the cholinergic deficit. The selective 5HT_4_ agonist prucalopride (Briejer *et al*. 2001) restored the impaired motility observed in TNX‐KO distal colon, an action known to be via increased cholinergic output (Leclere *et al*. 2005). Further studies are required to elucidate the mechanisms by which motility is specifically increased and the role of 5HT_4_. Of importance *in vitro* studies may not entirely reflect the natural state of the colon thus further *in vivo* studies will confirm whether impaired motility persists in TNX‐KO mice and indeed whether this can be rescued.

Reduction of distal colonic motility impairs both evacuatory function and colonic pellet‐forming motility, which we observed as reduced faecal output in TNX‐KO mice. Patients with TNX deficiency also demonstrated lower gut symptoms with significantly increased constipation and diarrhoea. Combined reports of constipation and diarrhoea could be due to a secondary problem in TNX‐deficient patients who start with severe constipation that often leads to bacterial overgrowth leading to reactive diarrhoea (Lokiec *et al*. 2014). Many patients in this study reported both diarrhoea and constipation, and thus have alternating bowel habits.

TNX‐deficient patients assessed in this study previously reported external rectal prolapse (Demirdas *et al*. 2016). Additionally, a history of rectal prolapse was most common in the hEDS subgroup compared to other EDS subtypes (Nelson *et al*. 2015). Similarity between human and mouse phenotype is clear, as TNX‐deficient mice also had internal rectal intussusception. Importantly, intussusception and prolapse are a logical consequence of inadequate control of lower GI smooth muscle.

A role of TNX in neural control of ion transport was suggested by its abundance in submucous neurones, many of which innervate epithelial cells. Activation of intrinsic enteric neurones to the mucosa releases non‐cholinergic transmitters, e.g. vasoactive intestinal peptide (VIP) and to a lesser degree ACh. These increase *I*_Sc_ which reflects epithelial secretion of chloride and fluid. There was no difference in basal or veratridine‐stimulated *I*_Sc_ between WT and TNX‐KO colon. There was, however, a tendency for the pattern and time course of response to shift towards more triphases in *I*_Sc_ during the response. We may have expected, like the motility results, reduced cholinergic secretory responses, especially given the preponderance of TNX in submucous cholinergic neurones. However, VIP‐containing secretomotor neurones account for 80% of all submucosal neurones in the mouse colon (Foong *et al*. 2014), so the contribution of ChAT‐containing cholinergic neurones towards neural regulation of colonic secretion is relatively small (as borne out by these and previous studies) (Hyland & Cox, 2005). Thus, the complex mucosal *I*_Sc_ responses in colonic tissues from TNX‐deficient mice are less likely to be affected, but there may be subtle changes in responses to nerve activation. To delineate if TNX has any role in barrier function, further studies are required, for example on the expression of tight junction proteins in the TNX‐KO mouse as well as permeability assessments.

The significant increase in PGP9.5‐IR neuronal endings in the TNX‐KO colonic mucosa is probably due to a change in sensory innervation evidenced by a larger increase in CGRP‐IR, a marker for nociceptive afferents (Robinson *et al*. 2004). Thus, in the absence of TNX, there is a change in innervation that may result in increased sensitivity to pain. This may be due to the anti‐adhesive properties of TNX (Valcourt *et al*. 2015). TNX induces a loss of adhesion through the p38 mitogen protein‐activated kinase (MAP) pathway, which is required for cell detachment (Rosen *et al*. 2002). Similarly, TNX in the ENS may be involved in signalling pathways that repel sensory endings from invading the colonic mucosa. Indeed, ECM glycoprotein tenascin/J1 or janusin, which shares structural homology with TNX, acts as a barrier for neurite outgrowth in the CNS (Schachner *et al*. 1994).

In human studies, increased nerve fibre outgrowth measured by neuron‐specific enolase (a general neuronal marker) in colonic mucosa of irritable bowel syndrome patients has been reported (Yu *et al*. 2012; Dothel *et al*. 2015). Of importance, expression of transient receptor potential vanilloid type‐1 (TRPV1) involved in mediating pain was increased in IBS patients which correlated with increased abdominal pain scores (Akbar *et al*. 2008). Specifically, a 3.5‐fold increase in TRPV1 was reported (Akbar *et al*. 2008) similar to the 3‐fold increase in CGRP observed in the TNX‐KO mucosa. This suggests that similar to IBS patients who have increased visceral pain and increased nociceptive nerves, TNX‐KO mice may also have visceral hypersensitivity. We observed this in an increased sensitivity of high‐threshold colonic afferents at rest and during phasic distensions in TNX‐KO mice. Other changes are possible, including changes to ion channel phenotype in sensory nerves that promote afferent depolarization (Jones *et al*. 2005; Yiangou *et al*. 2007). Increased nociceptive endings and hypersensitive afferents in TNX‐KO mice correlate with increased abdominal pain in TNX‐deficient patients reported in this study and in hEDS patients (Fikree *et al*. 2014) that is likely to constitute the increase in visceral pain. Of significance, molecules within the ECM interact with the β subunit of the voltage‐gated sodium channels (Na_v_) (Fuentes & Christianson, 2016) that regulate excitability of sensory neurones by reducing the activation threshold (de Carvalho Rocha *et al*. 2014). Further expression studies of TRP channels and voltage‐gated ion channels, for example Na_v_, would elucidate the role of TNX in visceral pain pathways. The oestrus cycle has potent effects on nociceptive pathways. Whether the differences we observed are complicated by altered endocrine function requires further study using male mice.

Tenascins were once labelled ‘talented proteins in search of function’ (Hsia & Schwarzbauer, 2005). Since then research has shown multiple roles for various tenascins. Our data identify the localization of TNX and its importance in colon, so we can relabel this molecule as a talented protein with at least two functions in the ENS. Of importance, our studies set the basis for exploring the precise mechanisms by which TNX modulates neuronal behaviour and function, which will set a framework for further investigations aimed at deciphering the role of ECM in gastrointestinal function and disease. Moreover, TNX genotype should be evaluated routinely in hEDS patients to better understand its role in specific symptoms, and thus allow targeted treatment of colonic motility (Lacy *et al*. 2009) and abdominal nociception (Lyubashina *et al*. 2015).

## Additional information

### Competing interest

The authors declare no competing financial interests.

### Author contributions

All authors had access to the study data and reviewed and approved the final manuscript. Professor Aziz and Professor Blackshaw are joint senior authors. Study concept and design‐ LAB, QA, RA. Acquisition of data‐ RA, MP, NCV, PW, VCG. Analysis and interpretation of data‐ RA, LAB, MP, VCG. Drafting of the manuscript‐ RA. Critical revision of the manuscript for important intellectual content‐LAB, QA RA. Statistical analysis‐ RA, MP. Obtained funding‐ LAB, QA. Administrative, technical or material support‐ AF, MW, IRT, VCG, EJA, SDM, JM, SMS, DB, HJC, NCV, CB. Study supervision‐ LAB, QA, NCV, EJA.

### Funding

This work was funded by a Wellcome Trust University Award and a Henry Smith Charity Grant to Prof. Blackshaw, a Bowel and Cancer Research PhD studentship to Dr Aktar, and an Ehlers Danlos Society Award to Prof. Aziz.
